# The Use of Intraoperative Radiography for Osteotomy Planning in Craniosynostosis Posterior Cranial Vault Distractor Application, Technique Description

**Published:** 2020-07-06

**Authors:** Taghreed Alhumsi, Abdulaziz Alshenaifi, Mohammed Almarghoub

**Affiliations:** ^a^Plastic Surgery Division, King Saud University Medical City, Riyadh, Saudi Arabia; ^b^College of Medicine, King Saud bin Abdulaziz University for Health Sciences, Riyadh, Saudi Arabia; ^c^Plastic Surgery Department, King Faisal Specialized Hospital & Research Centre, Riyadh, Saudi Arabia

**Keywords:** posterior cranial vault distraction, PVDO, craniosynostosis, cranial osteotomy, distractor application

## Abstract

Posterior cranial vault distraction osteogenesis (PVDO) is evolving as one of the first-line treatments in managing craniosynostosis. Intraoperative decision for sites of osteotomies requires precise planning and accurate placement of distractors. Objective: This technique helps in determining and confirming the pre-surgical planned osteotomy sites along with the distraction vectors. Technique: intraoperative plain skull radiography is used to determine the osteotomy site guiding placement of distractors using radio-opaque instrument. Result and discussion: we believe this technique helps translate the planned osteotomy sites and distraction vectors accurately to the skull and minimizes intra-operative error which will subsequently improve outcomes. Conclusion: This technique is a quick and safe tool for proper placement of posterior cranial vault distractor. However, further comparative studies are needed in addition to measuring cost, time added to the procedure, and radiation exposure.

Posterior cranial vault distraction osteogenesis (PVDO) is one of the options used to manage children with craniosynostosis. It is evolving as one of the first-line treatments in managing craniosynostosis with increased intracranial pressure.^1^ In addition, PVDO is believed to result in a greater increase in cranial volume than in fronto-orbital advancement.^2^ Multiple methods of distraction have been described, including single vector, multiple vectors, and spring-assisted distraction,^3^^,^^4^ all of which need thorough clinical and radiological presurgical planning to ascertain the best outcome.^5^ It has been noticed that intraoperative decision for the sites of osteotomy has been correlated with preoperative computed tomographic (CT) scans. We believe that the intraoperative calvarial dimension could be different from that predicted by CT scans, especially if the scans were dated much earlier than the surgical date. There are cases where it is difficult to correlate the CT scan to the intraoperative situation as planned preoperatively. Therefore, we suggest the use of intraoperative skull radiography as an available and straightforward technique that helps in determining and confirming the presurgical planned osteotomy sites along with the distraction vectors.

## TECHNIQUE

**Figure 1:**
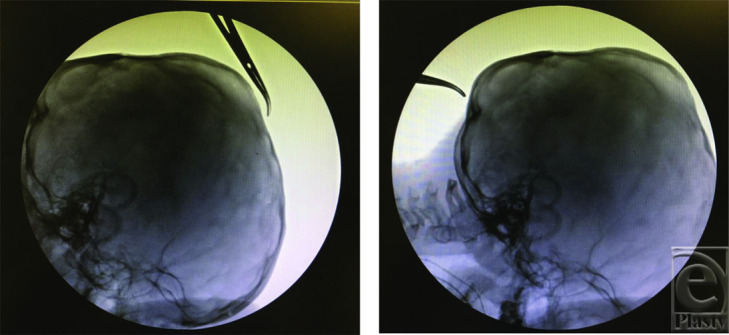
A towel clip is used to locate the future placement of the superior (left) and inferior (right) osteotomies at the beginning of the surgery to properly locate the bicoronal incision.

**Figure 2:**
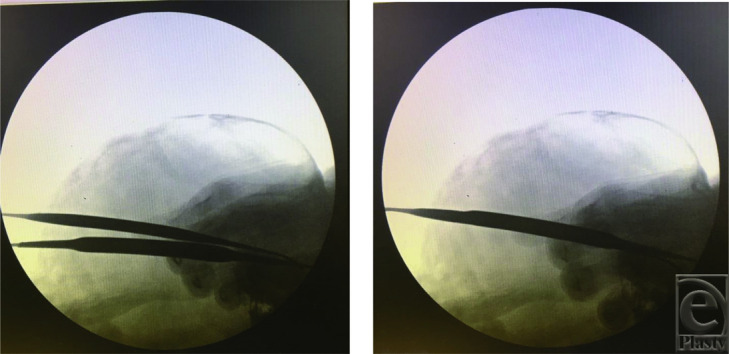
The superior and inferior osteotomy lines are marked using straight elevators under x-ray guidance. Elevators are adjusted (left) until they are parallel (right) before the osteotomy is done.

**Figure 3:**
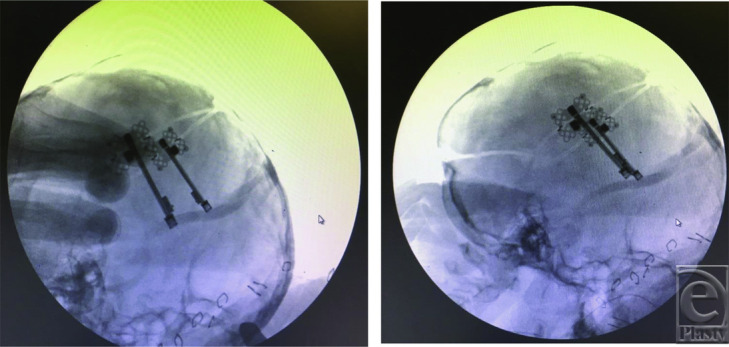
Right- and left-sided distractors (left) are adjusted to be parallel (right) in vector and share the same direction.

The patient is anesthetized and placed in a prone position with a mild neck extension and with careful attention paid to head support and ventilation tubes. After positioning the head, the hair is shaved completely. An initial lateral radiograph is then taken of the skull using a C-arm machine. A towel clip or any radiopaque instrument is used to locate the future placement of the superior osteotomy and the corresponding bicoronal incision under x-ray guidance (Fig 1). A bicoronal incision is designed in a zigzag fashion in accordance with the chosen site of the osteotomy earlier confirmed by a radiograph. The patient is then prepped and draped in a sterile fashion. A local anesthetic solution is used and injected subcutaneously, and locking hemostatic sutures are stitched on both edges of the anticipated bicoronal flaps to minimize bleeding. The flaps are elevated. After the elevation of the flaps, the superior and inferior osteotomy lines are marked using straight radio-opaque instruments, such as Kirschner wires or any straight instrument from the operative set, under x-ray guidance. The inferior osteotomy line is placed as inferior as possible to help expand the posterior vault and minimize step-off. Radiography helps make both right-sided and left-sided cuts parallel (Fig 2). We usually use 2 distractors, one on each side of the skull. The distractor positions, orientation, and vectors are also confirmed before cutting the osteotomy. It is essential to make sure that both right- and left-sided distractors are parallel in vector and share the same direction (Fig 3). Burr holes in preparation of the osteotomy are designed away from the distractor footplates. Distractors are then fixed accordingly, and a single drain is inserted in the posterior flap. The flaps are closed in a layered fashion with planned puncture-like openings that are created for each distractor arm on each side. Finally, a single x-ray shot is taken to confirm proper placement of distractors at the end of the surgery. The patient is extubated at the end of the procedure. X-ray protection garments and shields are used throughout the procedure.

## DISCUSSION

We reviewed several articles in the literature describing technical points in the application of PVDO. It was perceived that clinical and radiological presurgical planning is crucial to the success of the surgery. A vast majority of authors have described the use of preoperative CT and plain radiographs.^4^^,^^6^^,^^7^ Preoperative planning is crucial to decide distractor placement osteotomy lines and decrease complications arising from faulty distractor placement. These may include uneven distraction, unparallel vectors, and inferior step-off, which may affect skull contour.^4^ In addition, plain radiographs are used routinely during distraction activation and consolidation periods to assess distraction vectors, distraction progression, ossification, and patency of the distractor, which indicate the reliability of plain radiographs.

In addition to preoperative imaging, new methods of presurgical planning have emerged: computer-aided design (CAD) and 3-dimensional modeling. These methods enable surgeons to virtually apply osteotomies and direction vectors and auto-predict the outcomes.^5^^,^^8^ These methods were used in the pursuit of certain goals, including less operative time and primarily avoiding the chances of intraoperative trials and errors. Nevertheless, although these methods are showing promising outcomes, cost, radiation exposure, and preoperative time consumption are challenging barriers.^5^^,^^8^

In our center, all patients undergoing PVDO undergo CT scan as a part of their preoperative evaluation. This helps guide the expected osteotomy sites and distraction vectors easily. However, the intraoperative interpretation may be difficult due to many factors. Among those are (1) CT scan dimensions, (2) patient position during CT in comparison with surgery, and (3) surgical experience. We believe that using radiography intraoperatively is a readily available, cheap, rapid, and safe tool. It helps translate the planned osteotomy sites and distraction vectors accurately to the skull and minimizes intraoperative error. This also aids in correctly placing the inferior osteotomy line to optimize posterior vault expansion and minimize step-off. This improves the outcomes by confirming the site and vector. However, this may present itself as an extra step in the procedure with increased exposure to radiation for the patient, the surgeon, and the anesthesiologist. But it was reported that radiation exposure to C-arm fluoroscopy is considered below the maximum exposure limit in most orthopedic procedures, as they need a higher number of x-ray shots and even fluoroscopy than in our technique.^9^^,^^10^

PVDO and other methods of craniosynostosis surgical interventions must be thoroughly planned using clinical and radiological tools. Multiple methods are currently used, including CT scans and CAD. It was perceived that all previously mentioned methods are aiming for increased accuracy and decreased intraoperative errors. Therefore, we believe that confirming planned osteotomy sites and the position of vectors intraoperatively using radiography improves the outcome and vector direction in such procedures. We recommend applying this extra step in the procedure, as plain radiography is a quick and safe tool for this function, and then comparing the outcomes with the conventional technique in addition to measuring cost, time added to the procedure, and radiation exposure.
